# Body Mass Index: An Effective Predictor of Ejection Fraction Improvement in Heart Failure

**DOI:** 10.3389/fcvm.2021.586240

**Published:** 2021-12-01

**Authors:** Li-fang Ye, Xue-ling Li, Shao-mei Wang, Yun-fan Wang, Ya-ru Zheng, Li-hong Wang

**Affiliations:** ^1^Department of Cardiology, Zhejiang Provincial People's Hospital, Hangzhou, China; ^2^People's Hospital of Hangzhou Medical College, Hangzhou, China

**Keywords:** body mass index, obesity, left ventricular ejection fraction, improvement, heart failure

## Abstract

**Background:** Heart failure patients with higher body mass index (BMI) exhibit better clinical outcomes. Therefore, we assessed whether the BMI can predict left ventricular ejection fraction (EF) improvement following heart failure.

**Methods and Results:** We included 184 patients newly diagnosed with dilated cardiomyopathy and reduced EF in our center and who underwent follow-up examination of EF via echocardiography after 6 months. The EF improved at 6 months in 88 participants, who were included in the heart failure with recovered EF (HFrecEF) subgroup. Patients in whom the EF remained reduced were included in the heart failure with persistently reduced EF (persistent HFrEF) subgroup. Our analyses revealed that EF increase correlated with age (*r* = −0.254, *P* = 0.001), left ventricular diastolic dimension (LVDD; *r* = −0.210, *P* = 0.004), diabetes (*P* = 0.034), brain natriuretic peptide (*r* = −0.199, *P* = 0.007), and BMI grade (*P* = 0.000). BMI grade was significantly associated with elevated EF after adjustment for other variables (*P* = 0.001). On multivariable analysis, compared to patients with persistent HFrEF, those with HFrecEF had higher BMI [odds ratio (OR) = 2.342 per one standard deviation increase; *P* = 0.001] and lower LVDD (OR = 0.466 per one standard deviation increase; *P* = 0.001). ROC-curve analysis data showed that BMI > 22.66 kg/m2 (sensitivity 84.1%, specificity 59.4%, AUC 0.745, *P* = 0.000) indicate high probability of EF recovery in 6 months.

**Conclusions:** Our data suggest that higher BMI is strongly correlated with the recovered EF and that BMI is an effective predictor of EF improvement in patients with heart failure and reduced EF.

## Introduction

Heart failure (HF) is a serious health problem worldwide, with an estimated 1–2% of the adult population in the western world being affected by HF (1). In Europe, the estimated annual mortality rates for hospitalized and stable HF patients is 17 and 7%, respectively (2).

On the basis of left ventricular ejection fraction (EF), the current guidelines divide HF patients into three categories: (a) those with reduced ejection fraction (EF) (HFrEF; EF <40%), (b) those with preserved EF (HFpEF; EF ≥50%), and (c) those with a mid-range EF (HFmrEF; EF 40–50%) (3). Some studies indicated that the all-cause mortality rate in patients with HFrEF is generally higher than that in patients with HFpEF (2, 4, 5). For the management of HFrEF, device treatment is recommended after 3–6 months of medication to minimize the risk of all-cause mortality. Despite advances in HF management, the all-cause mortality rate in patients with this condition is high. Recent studies have indicated that some HFrEF patients, particularly those with dilated cardiomyopathy, may show substantial or even complete recovery of left ventricular systolic function with modern disease-modifying therapy; this correlates with a better patient outcome relative to persistent HFrEF (6–8). Therefore, it is essential to determine the indicative factors to identify HFrEF patients with a potential for cardiac recovery (HFrecEF) and those more likely to have persistent HFrEF who may benefit from even more aggressive therapies, such as implantable cardioverter defibrillators and cardiac resynchronization therapy (CRT).

Obesity is a significant public health concern in most countries (9). Previous studies have indicated that a high body mass index (BMI) significantly elevates the risk of cardiovascular disease and HF (10–12). However, in HF patients, being overweight or obese is associated with an better prognosis (13, 14). The clinical follow-up of HF patients with reduced EF revealed that higher BMI may be associated with a greater recovery of EF, especially in newly diagnosed dilated cardiomyopathy. However, the relationship between BMI and EF improvement in HFrEF is unclear. In this study, we evaluated whether BMI is an effective predictor of improved EF in dilated cardiomyopathy.

## Methods

### Study Population

We reviewed the medical records of adult patients diagnosed with dilated cardiomyopathy between September 1, 2017 and September 30, 2020 at the HF care department in our center. Patients meeting the following criteria were excluded from the study: (1) aged <18 years; (2) New York Heart Association (NYHA) class <I; and (3) missing data on the EF value in echocardiograms taken during the first visit or during the follow-up after 6 months. Next, we extracted data on baseline characteristics, including age, sex, laboratory test results, echocardiographic data, and comorbidities, from the patient medical records. BMI was calculated using the weight and height measurements taken at the first hospital visit. Where multiple echocardiograms had been taken over time, data from the 6 month follow-up visit were used to assess EF improvement. The study was performed in accordance with the international guidelines on clinical investigation of the World Medical Association's Declaration of Helsinki. As this was a retrospective study, no informed consent was required. The institutional review board approved the use of hospital records for the study.

### Diagnosis and Classification

Dilated cardiomyopathy is defined as left ventricular chamber dilation with decreased systolic function in the absence of hypertensive heart disease, cardiac valvular disease, congenital heart disease, ischemic heart disease, or conditions which impose a chronic pressure overload as based on the report of the World Health Organization/International Society and Federation of Cardiology 1995 classification of cardiomyopathies (15). Echocardiography was performed by experienced echocardiographers. The left ventricular diastolic dimension (LVDD), and EF were recorded. EF was calculated using Simpson's method in a four-chamber view. All measurements were performed using ultrasound systems (Philips EPIQ7C; Philips, indhoven, The Netherlands). BMI was calculated as both a continuous variable and a categorical variable. Subjects with values <18.5 were considered to be with underweight, those with values between 18.5 and 25.0, normal weight, those with values between 25.0 and 30.0, overweight, and those with values ≥30.0, obese (16).

To evaluate the association between BMI and EF recovery, we performed analyses that categorized the HFrEF into two classes depending on data from their follow-up echocardiograms. Patients with EF ≥40% were considered to have HFrecEF, according to the suggestion that these patients may be clinically distinct from those with persistently reduced EF (17, 18). The other participants were considered to have persistent HFrEF. In addition, follow-up EF ≥50% was used as an alternative definition of recovered EF for the second analysis.

### Statistical Analysis

The distribution of categorical variables was expressed in percentage, while continuous data was expressed as medians (interquartile range). Baseline characteristics between groups were compared using the chi-squared test for categorical variables. For continuous variables, the Mann-Whitney *U* test or Kruskal-Wallis test was used. Similar tests were used for subsequent analyses. The Spearman correlation and linear regression analysis were used to verify the association of EF changes with the related factors. Multivariable logistic regression analysis was used to evaluate the independent effect of the BMI on EF recovery at 6 months after diagnosis, with selected confounder parameters (significant association in univariate analysis). Receiver operating characteristic (ROC) curve was constructed to assess the discriminatory power of BMI for the EF recovery. The optimal cut-off point for BMI value for predicting EF recovery was performed by maximizing the Youden Index in a ROC curve analysis. *P*-value < 0.05 was considered statistically significant. Statistical analyses were done using SPSS version 19.0 (IBM Corp., Armonk, NY, USA).

## Results

### Baseline Clinical Characteristics

Of the 2,130 patients hospitalized for HF, 505 patients were diagnosed with dilated cardiomyopathy, and 309 patients met our cohort inclusion criteria of dilated cardiomyopathy diagnosed for the first time. Of these, 213 patients had a baseline EF of <40%, among whom 184 patients underwent a 6-month echocardiographic assessment of EF and were included in our analysis. The cohort comprised 78.8% male patients, and the patient age ranged from 22 to 91 years. The NYHA class in 86 (46.7%) and 60 (32.6%) patients was III and IV, respectively. After discharge, most patients were taking medications, including angiotensin converting enzyme inhibitors (ACEis), or angiotensin II receptor blockers (ARBs), or angiotensin receptor neprilysin inhibitors (ARNIs) (81.5%), mineralocorticoid receptor antagonist (MRAs; 87.0%), and/or beta blockers (88.0%). In all, 121 (65.8%) patients were on optimal medical therapy including ACEi/ARB/ARNI, Beta-blockers and MRA treatment simultaneously. No patient in our cohort had received CRT devices before inclusion. However, there were 30 patients with CRT implanted between the two echocardiograms.

### BMI and Change in EF

**Figure 1:**
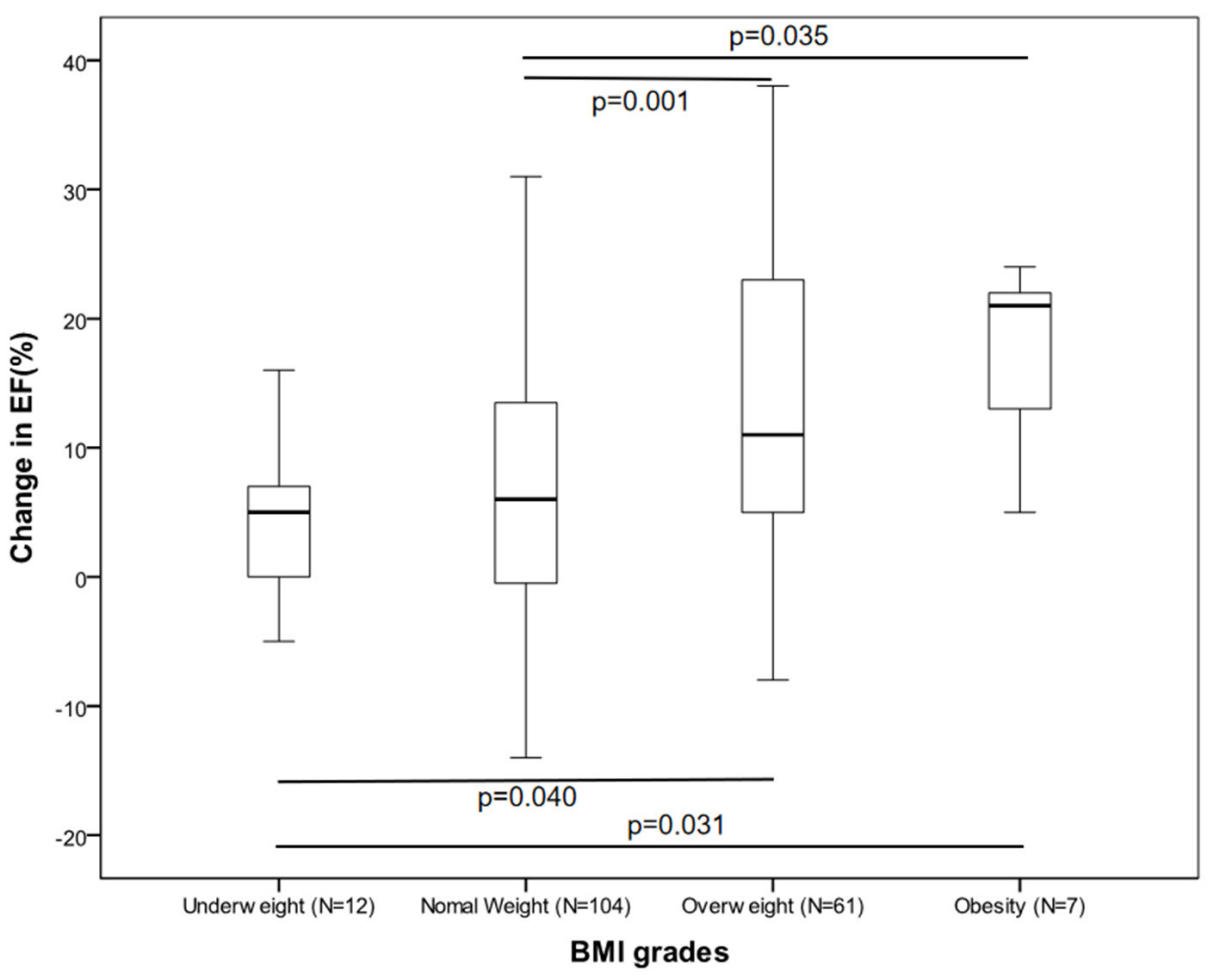
Box plot comparing change in ejection fraction by BMI group.

The participants' clinical features and their treatments according to their BMI categories are outlined in Table 1. At baseline, patients in the higher BMI group were younger compared with those in the lower BMI group. Plasma brain natriuretic peptide (BNP) and creatinine were very similar between the groups. There was little difference in the comorbidities and medication usage data. However, at 6 months, the obese and overweight patients had a significantly higher EF than those who were underweight or had normal weight. Analysis of the relationship between EF increases and the BMI group revealed that the change in EF were statistically significant when the four groups were analyzed together (Figure 1). After correcting for significance level, similar observations were made when the overweight group was compared to the other groups, but this did not apply to the obese group (underweight, *P* = 0.040; normal weight, *P* = 0.001).

**Table 1:** Clinical characteristics according to baseline body mass index.

**Variable**	**Underweight** **(*N* = 12)**	**Normal weight** **(*N* = 104)**	**Overweight** **(*N* = 61)**	**Obesity** **(*N* = 7)**	***P*** **-value**
Age (Years)	73.0 [67.5–76.6]	64.0 [55.3–72.8]	59.0 [45.0–72.0]	44.0 [29.0–50.0]	0.000
Male	9 (75.0)	81 (77.9)	48 (78.1)	7 (100.0)	0.564
Diabetes	2 (16.7)	14 (13.5)	15 (24.6)	2 (28.6)	0.284
Hypertension	2 (16.7)	41 (39.4)	33 (54.1)	3 (42.9)	0.072
Prior stroke	0 (0)	12 (11.5)	4 (6.6)	0 (0)	0.359
CAD	2 (16.7)	14 (13.5)	12 (19.7)	1 (14.3)	0.768
AF	2 (16.7)	36 (34.6)	16 (26.2)	1 (14.3)	0.346
LVDD (mm)	68.0 [61.3–75.8]	65.0 [62.0–71.8]	66.0 [61.0–70.0]	64.0 [61.0–68.0]	0.569
Creatinine (umol/l)	93.9 [77.8–127.1]	92.1 [77.1–110.2]	93.1 [77.8–111.6]	89.0 [81.3–106.9]	0.866
BNP (pg/ml)	1899.4 [785.7–3244.5]	706.9 [290.3–1595.4]	778.3 [329.3–1719.4]	508.2 [431.5–1182.6]	0.113
QRS (mm)	120.0 [100.0–158.5]	99.5 [85.0–137.5]	105.0 [90.0–145.0]	90.0 [85.0–105.0]	0.147
NYHA functional Class					0.522
II	2 (16.7)	25 (24.0)	11 (18.0)	0 (0)	
III	4 (33.3)	48 (46.2)	29 (47.5)	5 (71.4)	
IV	6 (50.0)	31 (29.8)	21 (34.4)	2 (28.6)	
ACEi/ARB/ARNI	11 (91.7)	81 (77.9)	51 (83.6)	7 (100.0)	0.321
Beta-blockers	9 (75.0)	90 (86.5)	56 (91.8)	7 (100.0)	0.269
MRA	11 (91.7)	91 (87.5)	53 (86.9)	5 (71.4)	0.626
Optimal medical therapy	8 (66.7)	63 (60.6)	45 (73.8)	5 (71.4)	0.379
Loop diuretics	12 (100.0)	92 (88.5)	55 (90.2)	6 (85.7)	0.641
Digoxin	9 (75.0)	63 (60.6)	34 (55.7)	1 (14.3)	0.062
CRT (between the two echos)	3 (25.0)	19 (18.3)	8 (13.1)	0 (0)	0.118
EF (%)					
Baseline	24.5 [21.0–31.5]	32.0 [28.0–36.0]	30.0 [27.0–35.0]	33.0 [28.0–36.0]	0.020
6-months	32.0 [22.3–38.5]	38.0 [31.0–43.0]	44.0 [36.5–55.0]	46.0 [43.0–57.0]	0.000
Change in EF	5.0 [−1.0–7.0]	6.0 [−0.8–13.8]	11.0 [5.0–23.5]	21.0 [9.0–23.0]	0.000
HFrecEF (%)	1 (8.3)	39 (37.5)	42 (68.9)	6 (85.7)	0.000

*CAD indicates coronary artery disease; AF, atrial fibrillation; LVDD, left ventricular diastolic dimension; EF, ejection fraction; BNP, brain natriuretic peptide; NYHA, New York Heart Association; ACEi, angiotensin converting enzyme inhibitor; ARB, angiotensin II receptor blocker; ARNI, angiotensin receptor neprilysin inhibitor; MRA, mineralocorticoid receptor antagonist; Optimal medical therapy, ACEi/ARB/ARNI and Beta-blockers and MRA simultaneously; CRT, cardiac resynchronization therapy.*

*P-values were obtained by using the Kruskal-Wallis test for continuous variables and the chi-square test for categorical variables*.

To further understand the impact of BMI on the change in EF at 6 months, we analyzed the correlation between characteristics and the EF increase 6 months after diagnosis (Table 2). These analyses found no significant relationship between EF increase and sex, hypertension, drug usage, or CRT. However, EF increase was correlated with age (*r* = −0.254, *P* = 0.001), LVDD (*r* = −0.210, *P* = 0.004), diabetes (*P* = 0.034), BNP (*r* = −0.199, *P* = 0.007), and BMI grade (*P* = 0.000). The relationship between EF change and BNP weakened when it was evaluated by linear regression analysis (*P* = 0.120). Of note, we found that BMI grade statistically correlates with EF change upon adjustment for other variables (*P* = 0.001).

**Table 2:** Factors associated with 6-month change in left-ventricular ejection fraction among all.

**Variables**	**Correlation coefficient**	***P*** **-value**	**Variables**	**Regression coefficient**	***P*** -value^*^
Age (years)	−0.254	0.001	Age (years)	−0.222	0.002
LVDD (mm)	−0.210	0.004	LVDD (mm)	−0.179	0.009
BMI grade		0.000^a^	BMI grade	0.235	0.001
Diabetes		0.034^a^	Diabetes	0.136	0.045
BNP (pg/ml)	−0.199	0.007	BNP (pg/ml)	−0.116	0.120
Beta-blockers		0.537^a^			
CRT		0.722^a^			

*BMI, body mass index; LVDD, left ventricular diastolic dimension; BNP, brain natriuretic peptide; CRT, cardiac resynchronization therapy.*

*P, Correlation analysis for continuous variables.*

a *P, Non-parametric Tests for categorical variables.*

* *P, Linear Regression analysis for factors associated with change in ejection fraction*.

### Independent Correlates of Recovery of Ejection Fraction

**Figure 2:**
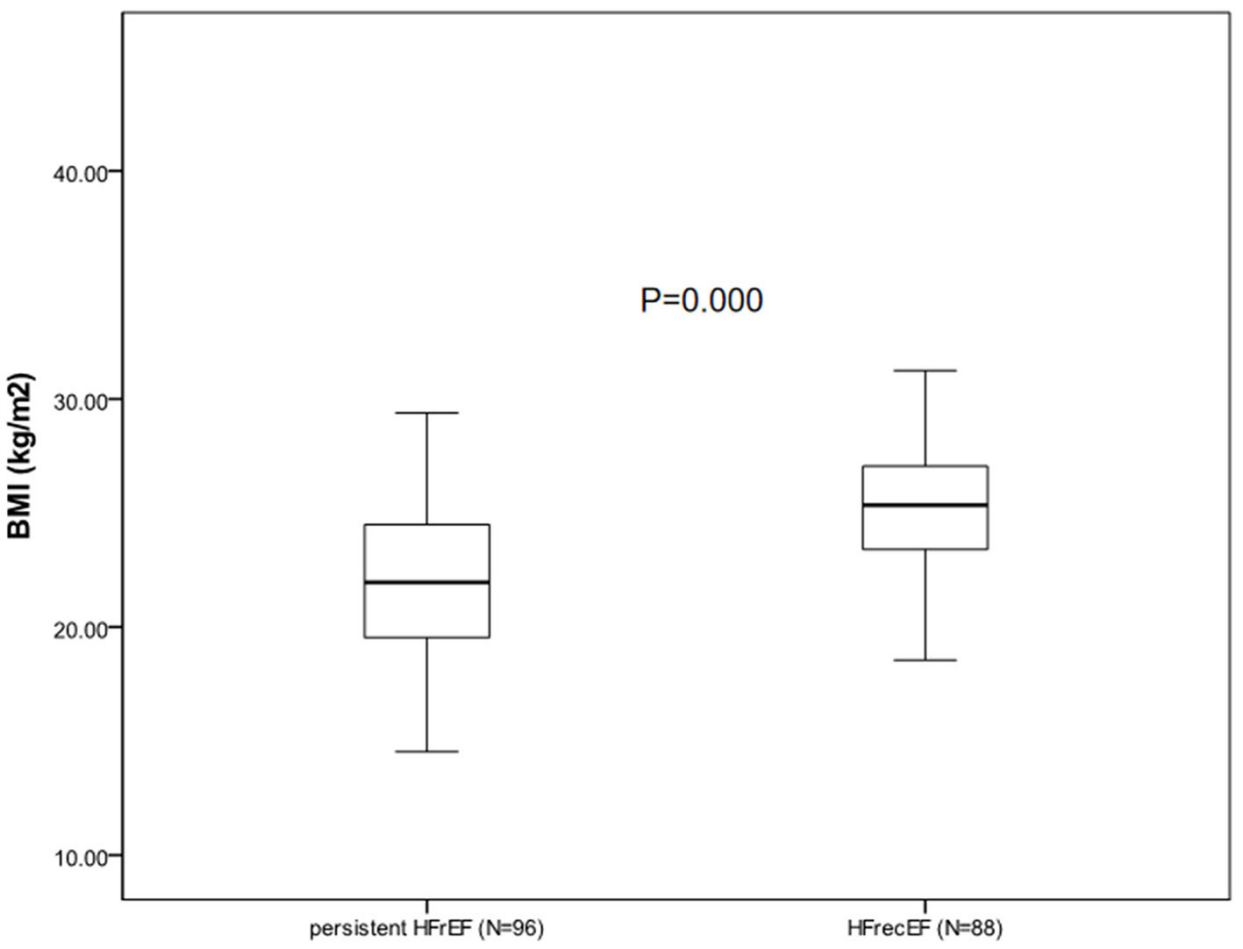
Box plot comparing BMI value between HFrecEF and persistent HFrEF group.

During the follow-up appointment after 6 months, 88 patients (47.8%) who had a 6-month EF of ≥40% were classified as having HFrecEF and 96 (52.2%) who had a 6-month EF of <40% were classified as having persistent HFrEF. Clinical features of patients with persistent HFrEF and HFrecEF at 6 months are shown on Table 3. BMI was managed as a consistent variable in the following analysis. At baseline, patients were younger and the BMIs were higher in the HFrecEF group than in the persistent HFrEF group. Individuals with coronary artery disease and atrial fibrillation had greater odds of falling in the HFrecEF group. However, differences in the presence of these comorbidities were not statistically significant. Treatments were very similar, although drug usage, including beta blockers, and MRAs, and CRT, were more frequent in persistent HFrEF patients. Our analysis indicated that diabetes mellitus or hypertension was more common in the HFrecEF group. And as a feature of HF, BNP was significantly higher in the persistent HFrEF group. In addition, the baseline EF values in the HFrecEF group was higher than in the persistent HFrEF group. During 6-month follow-up, the median EF improved from 33.5 to 47.0% in the HFrecEF group but remained low (median: 30.0–33.0%) in the persistent HFrEF group. The median EF change was 16% in patients with HFrecEF and 2% in patients with persistent HFrEF. It was observed that BMI was significantly higher in the HFrecEF group (*P* = 0.000). A box plot of BMI values for the different groups is shown in Figure 2.

**Table 3:** Clinical characteristics of patients between persistent HFrEF and HFrecEF group.

**Characteristics**	**HFrecEF (*N* = 88)**	**Persistent HFrEF (*N* = 96)**	***P*** **-value**
Male	68 (77.3)	77 (80.2)	0.626
Age (years)	60.0 [45.3–71.8]	65.5 [54.3–74.8]	0.008
BMI (kg/m^2^)	25.3 [23.4–27.1]	22.0 [19.5–24.5]	0.000
Hypertension	47 (53.4)	32 (33.3)	0.006
AF	33 (37.5)	22 (22.9)	0.031
CAD	15 (17.0)	14 (14.6)	0.647
Prior stroke	8 (9.1)	8 (8.3)	0.855
Diabetes mellitus	22 (25.0)	11 (11.5)	0.017
NYHA functional class			0.505
II	19 (21.6)	19 (19.8)	
III	44 (50.0)	42 (43.8)	
IV	25 (28.4)	35 (36.5)	
BNP (pg/ml)	588.1 [223.4–1111.8]	1157.6 [499.1–2393.9]	0.000
Creatinine (umol/l)	88.5 [77.8–107.5]	95.5 [80.0–113.0]	0.092
QRS (mm)	100.0 [85.8–120.0]	101.5 [90.0–149.8]	0.426
LVDD (mm)	63.5 [60.0–67.0]	68.0 [64.0–74.0]	0.000
ACEi/ARB/ARNI	71 (80.7)	79 (82.3)	0.779
Beta-blockers	77 (87.5)	85 (88.5)	0.828
MRA	73 (83.0)	87 (90.6)	0.123
Optimal medical therapy	57 (64.8)	64 (67.4)	0.711
Loop diuretics	76 (86.4)	89 (92.7)	0.158
Digoxin	50 (56.8)	57 (59.4)	0.725
CRT (between the two echos)	13 (14.8)	17 (17.7)	0.290
Baseline EF (%)	33.5 [28.0–36.0]	30.0 [27.0–35.0]	0.010
Second EF (%)	47.0 [43.0–55.0]	33.0 [26.5–37.0]	0.000
EF change (%)	16.0 [9.3–22.0]	2.0 [−3.0–6.0]	0.000

*EF, ejection fraction; Persistent HFrEF, heart failure with persistently reduced EF; HFrecEF, HF with recovered ejection fraction; BMI, body mass index; AF, atrial fibrillation; CAD indicates coronary artery disease; NYHA, New York Heart Association; BNP, brain natriuretic peptide; LVDD, left ventricular diastolic dimension; ACEi, angiotensin converting enzyme inhibitor; ARB, angiotensin II receptor blocker; ARNI, angiotensin receptor neprilysin inhibitor; MRA, mineralocorticoid receptor antagonist; Optimal medical therapy, ACEi/ARB/ARNI and Beta-blockers and MRA simultaneously; CRT, cardiac resynchronization therapy.*

*P-values were obtained by using the Mann-Whitney U test for continuous variables and the chi-square test for categorical variables*.

**Figure 3:**
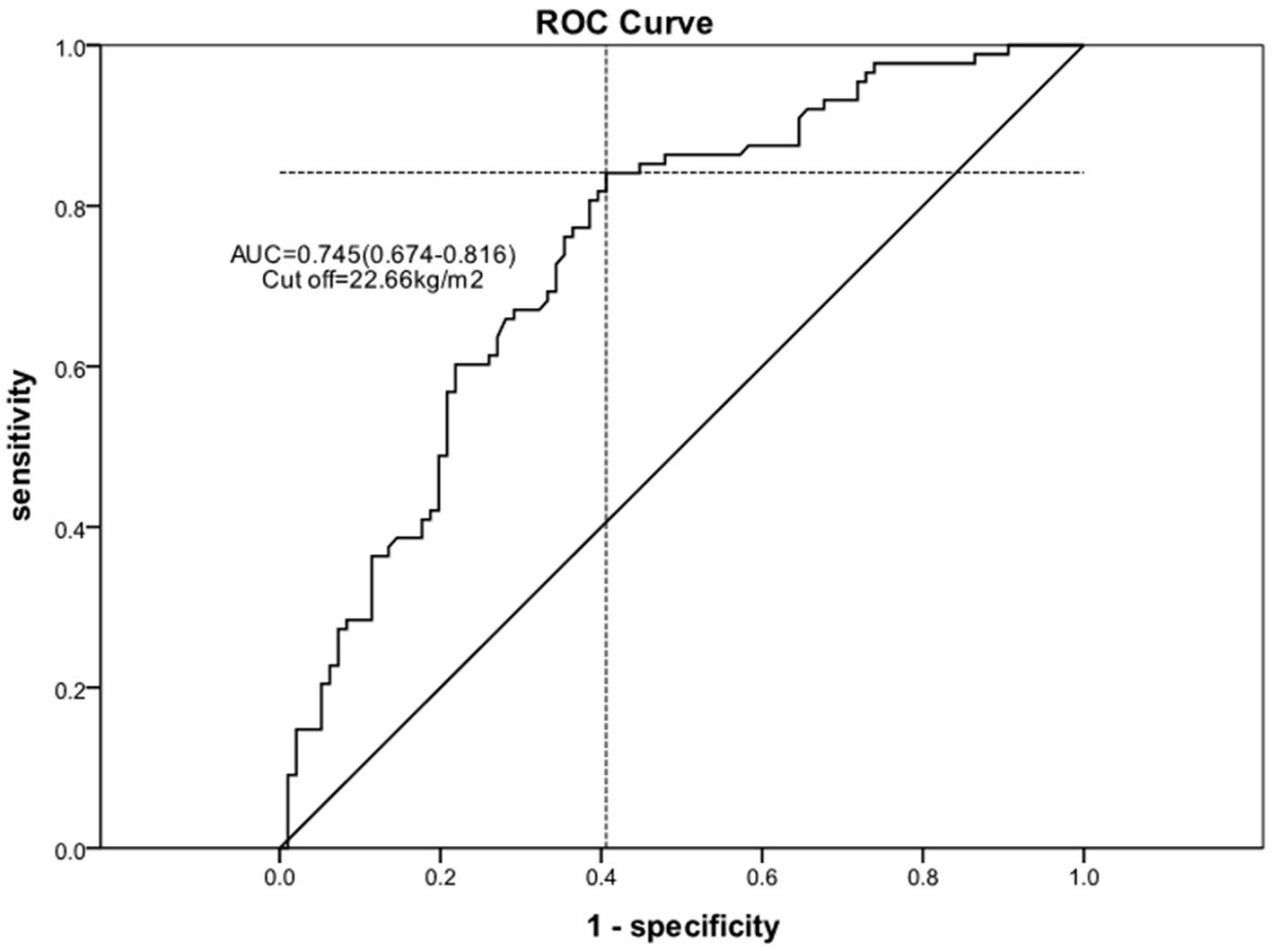
ROC curve for the cutoff value of the BMI.

Using logistic regression, univariate and multivariable predictors of absolute in EF recovery at 6 months were analyzed (Table 4). After adjustment for all other variables, BMI remained an independent predictor of absolute EF improvement [odds ratio (OR) = 2.342 per one standard deviation increase; 95% confidence interval (CI) 1.415–3.878; *P* = 0.001]. Other significant factors included LVDD (OR = 0.466 per one standard deviation increase; 95% CI 0.296–0.735; *P* = 0.001) and age (OR = 0.641 per one standard deviation increase; 95% CI 0.423–0.971; *P* = 0.036). We used the ROC curve analysis to elucidate BMI threshold values (Figure 3), in order to provide further evidence of the BMI as a predictor of EF recovery. ROC-curve analysis data showed that BMI > 22.66 kg/m^2^ (sensitivity 84.1%, specificity 59.4%, AUC 0.745, *P* = 0.000) indicate high probability of EF recovery in 6 months.

**Table 4:** Logistic Regression Analysis of baseline patient characteristics associated with HFrecEF (vs. persistent HFrEF).

**Variables**	**Univariate**		**Multivariable**	
	**OR (95%CI)**	***P***	**OR (95%CI)**	***P***
Age (years)^*^	0.625 (0.459–0.852)	0.003	0.641 (0.423–0.971)	0.036
BMI (kg/m^2^)^*^	2.804 (1.840–4.272)	0.000	2.342 (1.415–3.878)	0.001
BNP (pg/ml)^*^	0.555 (0.393–0.782)	0.001	0.715 (0.477–1.073)	0.105
LVDD (mm)^*^	0.433 (0.298–0.630)	0.000	0.466 (0.296–0.735)	0.001
Hypertension	0.436 (0.240–0.792)	0.006	0.876 (0.411–1.865)	0.731
Diabetes mellitus	0.388 (0.176–0.857)	0.019	0.392 (0.150–1.024)	0.056
AF	0.495 (0.261–0.942)	0.032	0.472 (0.205–1.084)	0.077
Baseline EF (%)	1.492 (1.100–2.023)	0.010	1.257 (0.848–1.863)	0.255

*BMI, body mass index; BNP, brain natriuretic peptide; LVDD, left ventricular diastolic dimension; AF, atrial fibrillation; EF, ejection fraction.*

* *Per one standard deviation increase was used for OR calculation*.

### Alternative Definition of Recovered EF

When EF at or above 50% was used to define recovered EF, 32 of 184 patients (18.4%) had a recovered EF at the 6-month visit. There were no statistically significant differences in comorbidities or therapies between groups. It was observed that BMIs were significantly higher in the HFrecEF group (*P* = 0.000), whereas age were higher in patients in the persistent HFrEF group. On multivariable analysis (Table 5), the factor that had a significant association with EF recovery was the BMI value (OR = 1.750 per one standard deviation increase; 95% CI 1.124–2.724; *P* = 0.013), apart from age (OR = 0.680 per one standard deviation increase; 95% CI 0.443–1.042; *P* = 0.077).

**Table 5:** Logistic Regression Analysis: predictors of recovery in left-ventricular ejection fraction (when EF ≥ 50% was used to define recovered EF).

**Variables**	**Univariate**		**Multivariable**	
	**OR (95%CI)**	***P***	**OR (95%CI)**	***P***
Age (years)^*^	0.535 (0.363–0.789)	0.002	0.680 (0.443–1.042)	0.077
BMI (kg/m^2^)^*^	2.061 (1.369–3.102)	0.001	1.750 (1.124–2.724)	0.013
LVDD (mm)^*^	0.638 (0.407–1.001)	0.051		

*BMI, body mass index; LVDD, left ventricular diastolic dimension; EF, ejection fraction.*

* *Per one standard deviation increase was used for OR calculation*.

## Discussion

Obesity is associated with an increased risk of HF. Curiously, obesity has also been proven to improve the HF prognosis (12). However, it has been unclear whether the BMI was related to EF improvement. Here, we sought to elucidate the predictors of EF recovery and the role of the BMI on EF improvement.

Our 6-month follow-up of patients hospitalized for HF showed that obese and overweight patients had similar impacts in that they were both more likely to have EF increases relative to patients with normal or underweight. Taken together, these data suggested the existence of an optimal BMI, which was beneficial for people with HFrEF. Similar observations were made after the analysis of BMI as a persistent variable. We indicated that higher BMIs to a certain degree were associated with a greater likelihood of EF improvement in HFrEF patients. This study highlighted the potential of using BMI as a predictor of EF improvement, and that patients with high BMI may have exhibit improved EF. Although, hypertension, atrial fibrillation, and diabetes mellitus associated with increased likelihood of recovered EF on univariate comparisons, neither were independently associated in the multivariable analysis. This can be explained by the fact that these factors are more common in cases with higher BMI which could predict ejection fraction recovery. Rhythm control of AF could also result in improvement in LVEF. Ghimire et al. recently reported similar result on the relation of hypertension and atrial fibrillation with recovered EF (19). Additionally, our data confirmed that basic LVDD was an important factor in EF recovery. We suggested that lower LVDD values were associated with higher EF recovery, which is consistent with previous reports (20). When we performed a secondary analysis using EF ≥50% as the cutoff for HFrecEF, the association of BMI and EF recovery was similar to that observed with the original definition.

Our data indicated that EF recovery was possible with guideline-directed treatment in a substantial proportion of patients with HFrEF. We included newly diagnosed dilated cardiomyopathy. The cutoff threshold for improved EF after HF was set at EF ≥40%, based on the mentions in several studies. The definition of improved EF and time intervals between echocardiograms varies widely across studies (17, 18, 21, 22). In this study, 47.8% of inpatients had recovered EF at the 6-month follow-up. However, the prevalence of HFrecEF is different based on the methodology in the study. A previous a single-center, observational study reported that 30.8% of patients with dilated cardiomyopathy recovered in 6 months (23). That study also included newly diagnosed dilated cardiomyopathy patients. While HFrecEF was defined as an EF elevation of ≥50% based on echocardiography. The definition of HFrecEF is different from the one in this study, therefore, more stringent than ours, which may account for the lower rate in patients with recovered EFs.

Our data indicated that patients with HFrEF and higher BMI are more likely to exhibit EF recovery. There are multiple explanations for this. First, patients with high BMIs tend to have higher muscle mass and metabolic reserves in the form of fatty tissue (24). Additionally, the symptoms appear at an earlier stage of HF in overweight or obese patients. This is likely due to lower strength and comorbidities frequently seen in overweight individuals, which may cause them to seek medical attention sooner. In such instances, the disease often adequately treated. Furthermore, patients with increased BMI exhibit increased levels of serum lipoproteins, which have anti-inflammatory effects and may, therefore, contribute to a decreased risk of mortality (25).

Recently, significant efforts has been taken to elucidate predictors of better outcomes in HF. It has been previously reported that being overweight or obese correlates with a better HF prognosis (26–28). Studies on adult HF with recovered EF found that patients with improved EF have more favorable outcomes than patients with persistent reduced EF (6, 19, 29). A meta-analysis of 48 studies found that age, renal function, EF, and BMI are strong predictors of HF mortality (30). Additionally, it has been shown that BMIs are higher in HFrecEF patients, which is consistent with our observations (31). However, none of these studies assessed the entire range of BMI values or BMI classifications and how the BMI influenced EF changes. To learn more about the subset of patients with recovered EF, we sought to elucidate the predictors of EF improvement and the role of BMI on EF recovery. This would help us judge the impact of BMI on EF improvement more precisely. However, we were unable to determine if an increased BMI resulted in a better outcome in HF directly as EF is just one of the factors influencing HF outcomes (30).

To the best of our knowledge, this is the first study to assess BMI in patients with dilated cardiomyopathy and report that overweight or obese patients are more likely to exhibit EF improvement, while patients with low or normal weight are more likely to have persistent HFrEF. We believed that patient with low BMI, would benefit from early aggressive therapies. We only included patients with newly diagnosed HF and dilated cardiomyopathy to minimize the impact of cachexia caused by advanced HF and eliminate the influence of different etiologies. The use of optimal medical therapy was lower in the our population compared with that seen in other clinical trials (6, 29). These might influence the proportion of patients with recovered EF as optimal medical therapy strongly affected reverse remodeling. In addition, the limitations of our study include the small participant size; incomplete data on the accurate dose of beta blockers or ARNIs; the lack of information on kidney disease, and lung disease; and the possibility of the BMI being overestimated in patients with edema at the onset of HF. BMI may, therefore, have changed during the follow-up phase, resulting in misclassification. These factors may have a different effect on the association between body weight and HFrecEF. The mortality rate was too low for analysis over a short follow-up period, and our analysis could not establish an association between BMI and survival. Further, we consider the change in BMI as a possible predictor for EF change. Therefore, larger prospective studies should be conducted to validate our study findings.

As stated above, the influence of BMI on EF change is particularly pronounced in patients with dilated cardiomyopathy, suggesting that BMI can be used to predict which patients are likely to exhibit improved EF. Additionally, this finding suggests that strategies promoting optimal body weight may improve cardiac function during HF and, therefore, improve prognosis.

## Conclusions

In this study of patients with newly diagnosed dilated cardiomyopathy and HF, we found that higher BMI and lower LVDD to a certain extent is significantly correlated with increased EF during the 6 months of follow-up. Our findings indicate that BMI is an effective tool to help predict EF changes in patients with dilated cardiomyopathy and reduced EF.

## Data Availability Statement

The raw data supporting the conclusions of this article will be made available by the authors, without undue reservation.

## Ethics Statement

The studies involving human participants were reviewed and approved by the Ethics Committee of Zhejiang Provincial People's Hospital. Written informed consent for participation was not required for this study in accordance with the national legislation and the institutional requirements.

## Author Contributions

L-fY: acquisition of data and rafting the article. X-lL and S-mW: analysis and interpretation of data. Y-fW and Y-rZ: revising it critically for important intellectual. L-hW: final approval of the version to be submitted. All authors contributed to the article and approved the submitted version.

## Funding

This study was supported by the National Natural Science Foundation of China (No. 81670447), Zhejiang Provincial Natural Science Foundation of China (No. LY15H020006), Zhejiang Province Key Subject of Medicine (Neurological Rehabilitation), the Medicine and Health Project of Zhejiang Province (No. 2017KY015) and the Traditional Chinese Medicine Program of Zhejiang Provincial (No. 2017ZZ001, No. 2017ZB005). LW was sponsored by Zhejiang Provincial Program for the Cultivation of High-Level Innovative Health Talents.

## Conflict of Interest

The authors declare that the research was conducted in the absence of any commercial or financial relationships that could be construed as a potential conflict of interest.

## Publisher's Note

All claims expressed in this article are solely those of the authors and do not necessarily represent those of their affiliated organizations, or those of the publisher, the editors and the reviewers. Any product that may be evaluated in this article, or claim that may be made by its manufacturer, is not guaranteed or endorsed by the publisher.
